# Mechanisms of pediatric ischemic strokes in COVID-19: a systematic review

**DOI:** 10.3389/fstro.2023.1197714

**Published:** 2023-07-03

**Authors:** Elbert John V. Layug, Almira Doreen Abigail O. Apor, Rudolf V. Kuhn, Marilyn A. Tan

**Affiliations:** ^1^Department of Neurosciences, College of Medicine and Philippine General Hospital, University of the Philippines Manila, Manila, Philippines; ^2^Department of Pediatrics, College of Medicine and Philippine General Hospital, University of the Philippines Manila, Manila, Philippines; ^3^National Kidney and Transplant Institute, Quezon City, Philippines

**Keywords:** COVID-19, pediatric stroke, childhood ischemic stroke, SARS-CoV-2, stroke in the young

## Abstract

**Background:**

Coronavirus disease 2019 (COVID-19) has been shown to cause vasculopathic and hemostatic derangements predisposing to cerebrovascular and thrombotic disorders in adults. Data in children, however, are limited to case reports and series. Given the unique risk factors and potential pathomechanisms in children, it is imperative to characterize stroke in children with COVID-19. Understanding these mechanisms is essential in drafting an appropriate management protocol to improve outcomes in a population where stroke carries higher disability-adjusted life years.

**Methods:**

A systematic literature search was done in MEDLINE, EMBASE, Web of Science and Google Scholar using the terms “pediatric ischemic stroke,” “cerebral sinovenous thrombosis,” “SARS-CoV-2,” and “COVID-19.” Patient demographics, clinical profile, stroke risk factors, neuroimaging findings, interventions and outcomes were recorded.

**Results:**

The search produced 776 records. After preliminary review of titles, abstracts and selected full texts, 52 articles comprising of 74 patients were studied. The cohort has slight female predominance (51.5%), with mean age of 9.2 years (±2SD 5.6). Pediatric ischemic strokes were categorized as arterial ischemic strokes (82.40%), cerebral sinovenous thrombosis (12.20%) and combined arterial and venous strokes (5.41%). Mechanisms of ischemic stroke included thrombophilia (47.3%), vasculopathies (27%) and cardioembolism (6.8%). Twenty cases (27%) had comorbidities predisposing to stroke and only 18.9% met the criteria for multisystem inflammatory syndrome in children (MIS-C). Outcomes ranged from complete recoveries (13/58), residual deficits (35/58), and mortalities (10/58).

**Conclusion:**

This study presents a comprehensive summary of the currently available published literature on pediatric ischemic strokes in the background of COVID-19. The clinical profiles and outcomes of patients reviewed support prior hypotheses that the virus can cause both a vasculopathy and induce a derangement in the coagulation system, predisposing to ischemic strokes.

**Study registration:**

This paper's protocol has been registered in PROSPERO with ID number CRD42022315219.

## 1. Introduction

The COVID-19 pandemic has led to one of the most challenging times in the healthcare system over the past century. Knowledge about this disease has increased exponentially through the years.

Currently, infection with SARS-CoV-2 is reported to cause a multitude of systemic manifestations and one of which is its deleterious effects on the nervous system. Either through direct invasion of the virus (neurotropism), or indirect effects on other organ systems, the nervous system may consequentially become a major target organ in this condition (Iadecola et al., 2020).

COVID-19-associated ischemic strokes have been well-described in adults and is partly due to the novel coronavirus' effects on the cardiovascular, hematologic and immunologic systems (Tan et al., 2020). In pediatrics, there is limited data on ischemic strokes occurring in COVID-19. Mostly, children who are exposed to SARS-CoV-2 undergo an asymptomatic or a less severe course of the illness in contrast to adults (Liguoro et al., 2020; Dawood et al., 2022).

In a multinational study done during the outset of the pandemic (March to May 2020), only 0.82% (8/971) of pediatric patients with SARS-CoV-2 had ischemic strokes. On the other hand, among confirmed cases of ischemic stroke, the proportion of those with COVID-19 was also very small ranging from zero for the 33 cases of neonatal cerebral sinovenous thrombosis (CSVT) to 3.6% of the reported 166 cases of childhood arterial ischemic strokes (AIS) (Beslow et al., 2021).

Through time, reports of pediatric stroke cases in the background of COVID-19 have accumulated in literature. Thus far, the available data has been restricted to case reports, series and retrospective cohort studies. In order to further understand the clinical burden and mechanisms of COVID-19-associated pediatric ischemic stroke, we performed a systematic review to evaluate the clinical characteristics, neuroimaging findings, risk factors, treatment and outcomes of pediatric patients with COVID-19 who have suffered ischemic strokes.

## 2. Methods

A systematic review utilizing the outlined approach in the Cochrane Handbook Systematic Reviews (Cochrane Training, 2022) was done. The collected data were reported using the Preferred Reporting Items for Systematic Reviews and Meta-analyses (PRISMA) (Page et al., 2021).

### 2.1. Criteria for study selection

Articles published from the date of inception of the databases until February 2023 were eligible. Criteria for inclusion included: (1) studies with methods described as case reports/series, case-control, cross-sectional, cohort, or randomized-controlled trials and (2) studies reporting pediatric patients with AIS or CSVT and COVID-19. Studies with insufficient data on patient demographics, stroke diagnosis or COVID-19 status and those without available full texts for review or English translation were excluded.

### 2.2. Search methods for identification of studies

A systematic search in MEDLINE (through PubMed), EMBASE, Web of Science and Google Scholar was done. Relevant search terms such as pediatric stroke, arterial ischemic stroke, cerebral sinus venous thrombosis and COVID-19 were used in the different search strategies. The comprehensive strategies for different databases are provided in the Supplemental Table 1.

### 2.3. Selection of studies

After generating results from the database search, duplicate entries were removed. Titles and abstracts that met the inclusion criteria were gathered for full review. Likewise, abstracts that did not provide satisfactory data concerning the mentioned criteria were incorporated for comprehensive text evaluation. Subsequently, two authors (EJVL and RVK) independently appraised the articles and completed their choices following the aforementioned criteria. Disagreements were decided by consensus or by the senior reviewer (MAT).

### 2.4. Risk of bias assessment

The quality of the included case reports and series were assessed utilizing the parameters suggested by the Johanna Briggs Institute. On the other hand, the observational cohort studies were evaluated utilizing the Newcastle-Ottawa scale.

Case reports were reviewed for the following data: patient demographics, clinical and neurological manifestations, pertinent diagnostics, treatment and outcomes. Likewise, case series were evaluated if there was mention of clear inclusion criteria, if conditions were reliably measured and with reported demographics, clinical data and outcomes. For the observational cohort study, it was evaluated based on its selection, comparability and outcomes.

### 2.5. Data extraction and outcome measures

The quantitative data were recorded using absolute numbers per event. The following information were retrieved using piloted data forms: authors, demographic information of the patients, comorbidities, neurologic manifestations, interval from stroke symptom onset to diagnosis, National Institutes of Health Stroke Scale (NIHSS) score, COVID-19 symptoms, neuroimaging findings, relevant diagnostics for etiology (e.g., echocardiogram, inflammatory markers), intervention done (thrombolysis, thrombectomy, anticoagulation, antiplatelet therapy) and outcomes.

Pediatric ischemic stroke type is categorized into neonatal AIS or CSVT and childhood AIS or CSVT. Identified pathomechanism of the stroke is classified as either (1) thrombophilia, (2) vasculopathy, or (3) cardioembolism.

Data were summarized using measures of central tendency for continuous variables. On the other hand, categorical data were summarized by presentation of percent distribution or frequencies.

## 3. Results

### 3.1. Included studies

**Figure 1:**
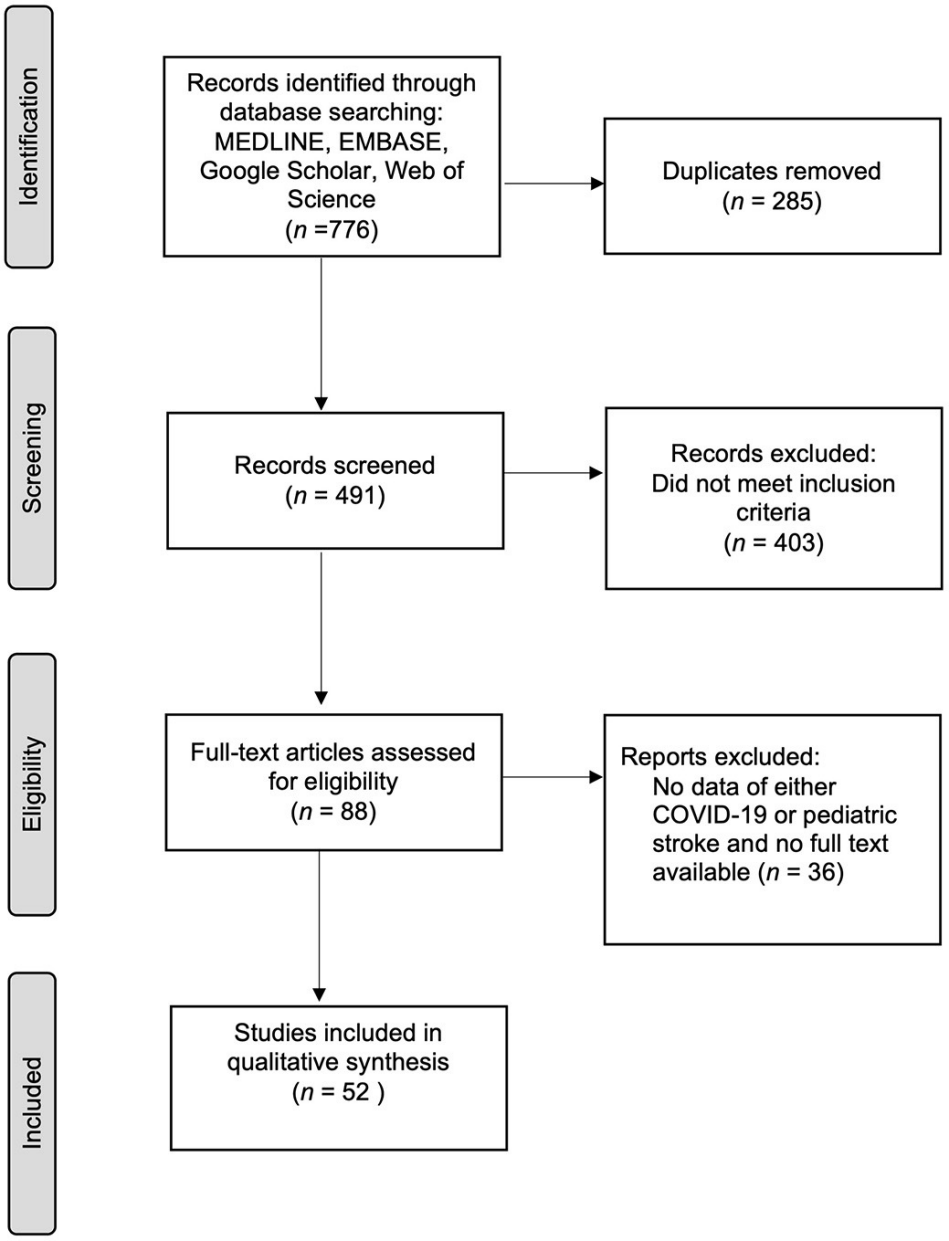
Summary of search and selection of articles.

Upon search in databases, 776 publications were identified. From which, duplicates were removed (*n* = 285) yielding 491 records. From these, 403 articles were excluded for not meeting the inclusion criteria. Subsequently, 88 publications passed for full text review. From this, 36 were eliminated for not meeting the set criteria. Upon detailed review, a total of 52 articles documenting 74 cases met the abovementioned criteria. These were incorporated in the qualitative analysis (Figure 1).

### 3.2. Patient demographics of the included population

The review included 74 pediatric patients diagnosed with ischemic strokes and COVID-19 (Supplemental Table 2). Majority of the sample population came from the United States of America (43%), India (13%), Iran (6%) and United Kingdom (6%). Clustering of cases were likewise noted from the Czech Republic (4.5%), Africa (4.5%), Italy (3%), Mexico (3%), Moldova (3%) and Turkey (3%). Single case reports were gathered from Bulgaria, France, Israel, Kuwait, Poland, Romania and Russia.

The cohort has female predominance (35/68, 51.5%), with mean age of 9.2 years old (±2SD 5.6; Range: 4 days−18 years old). COVID-19 was diagnosed in these patients via SARS-CoV-2 PCR test (26/74, 35.1%), serologic testing (30/74, 40.5%), or both (9/74, 12.2%). Nine of the reported confirmed cases had unspecified testing done.

### 3.3. Clinical profile of cases with ischemic strokes and COVID-19

#### 3.3.1. Ischemic strokes

**Figure 2:**
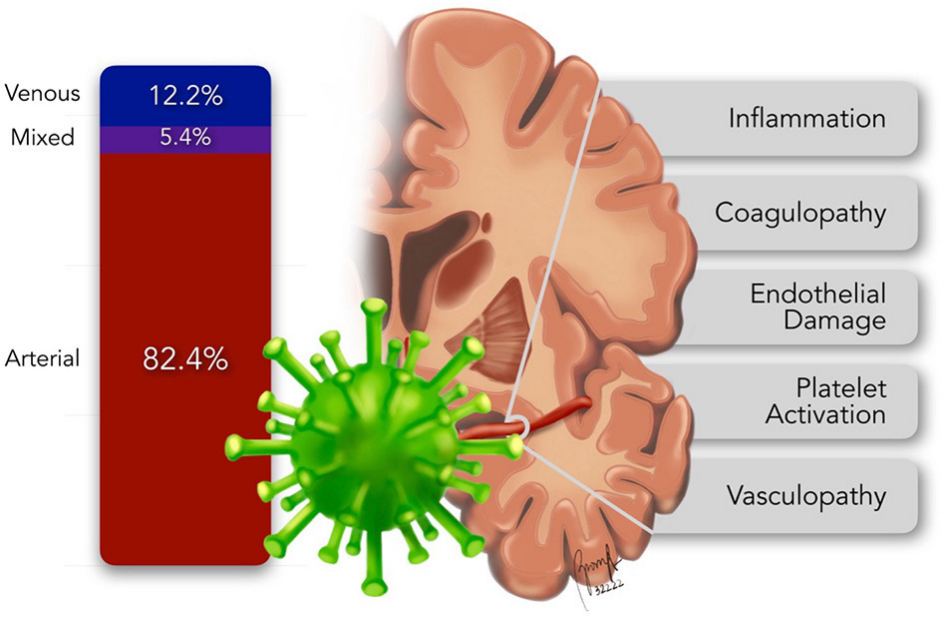
Distribution and pathomechanisms of pediatric ischemic stroke.

Majority (61/74, 82.4%) of the subjects were diagnosed with arterial ischemic strokes. On the other hand, 9/74 or 12.2% had cerebral venous thrombosis while 4/74 or 5.4% had combined arterial and venous strokes (Figure 2).

In arterial ischemic strokes, 47/62 or 75.8% of the cases involved the anterior circulation, 8/62 or 12.9% affected the posterior circulation while 7/62 or 11.3% involved both anterior and posterior circulation. Mechanisms of ischemic stroke included thrombophilia (35/74, 47.3%), vasculopathies (20/74, 27%) and cardioembolism (5/74, 6.8%). Timing of stroke ictus in relation to the COVID-19 symptoms and the timing of stroke-targeted intervention were not specified in the majority of the reviewed publications.

#### 3.3.2. Concomittant non-neurologic symptoms

In this cohort, 25/74 or 33.8% of the cases have concomitant non-neurologic or systemic symptoms at the time of the stroke ictus such as fever, myalgia or body malaise (23/25, 92%), respiratory (10/25, 40%) and gastrointestinal symptoms (5/25, 20%). Half of these cases had elevated inflammatory markers such as C-reactive protein, erythrocyte sedimentation rate, ferritin and D-dimer. This COVID-19-symptomatic subgroup did not meet the criteria for the diagnosis of multisystem inflammatory syndrome in children (MIS-C).

#### 3.3.3. Multisystem inflammatory syndrome in children

The MIS-C-confirmed symptomatic patients comprises 20/74 or 27% of the cohort. As per case definition, such patients had evidence of recent or current SARS-CoV-2 infection, fever, increased laboratory markers of inflammation, at least two target organ dysfunction without alternative plausible cause and were critically-ill requiring hospitalization (WHO, 2022b). Cardiac, renal, respiratory, gastrointestinal and neurologic involvement were reported in our data.

#### 3.3.4. Asymptomatic cohort prior to stroke ictus

On the other hand, 11/74 or 14.8% of patients had no past or present symptoms identifiable with COVID-19 until the stroke ictus. Three of these patients had elevated inflammatory markers at the time of evaluation.

Furthermore, although asymptomatic at presentation, twelve patients had constitutional, respiratory or gastrointestinal symptoms that resolved prior to stroke ictus. Such symptoms were most commonly reported at 7 and 30 days (Range: 7–60) prior to onset of the stroke. Of these seemingly post-infectious cases, half had elevated inflammatory markers.

#### 3.3.5. Cases with other comorbid illnesses

Twenty of the 74 cases or 27% had concomitant conditions known to be risk factors for stroke. These included other infectious causes (i.e., bacterial and tuberculous meningitis, Lemierre syndrome and malaria), cardiac anomalies, malignancy and hemoglobinopathies. Five cases were undergoing extracorporeal membrane oxygenation (ECMO) at the time of stroke.

### 3.4. Interventions done and outcomes

Stroke-directed therapeutic interventions in this cohort included thrombolysis (*n* = 5), mechanical thrombectomy (*n* = 5), use of antiplatelet agents such as aspirin (*n* = 24) and clopidogrel (*n* = 1), and anticoagulants including heparin (*n* = 9), enoxaparin (*n* = 14), LMWH (*n* = 8) and acenocoumarol (*n* = 1). Patients with documented vasculitis were given steroids (*n* = 24), other immunomodulators like rituximab (*n* = 1), tocilizumab (*n* = 1), infliximab (*n* = 1) and IVIG (*n* = 10 and calcium channel blocker like nimodipine (*n* = 1). On the other hand, management of COVID-19 included anti-viral medication like remdesivir (*n* = 5) and ECMO (*n* = 5) for the respiratory failure.

Majority (58/74) of the articles reviewed contained details on patient outcomes. Among these, 13/58 or 22.41% had complete resolution of the neurologic symptoms whereas 35/58 or 60.34% were discharged with residual deficits. The remaining patients (10/58, 17.24%) succumbed from either the critical course of the COVID-19 (5/10, 50%) or the catastrophic sequelae of the stroke (5/10, 50%).

## 4. Discussion

### 4.1. COVID-19 and the pathogenesis of ischemic strokes

Since the beginning of the COVID-19 pandemic, knowledge of the disease has continuously progressed. Although with less risk of exposure and getting screened than adults, the incidence of COVID-19 infection in children is similar to adults (Dawood et al., 2022). Despite having mostly asymptomatic infections (Dawood et al., 2022) and milder clinical courses (Liguoro et al., 2020; Mehta et al., 2020), children may have manifestations comparable to those of adults. These include constitutional, respiratory, gastrointestinal, dermatologic, cardiovascular, hematologic and neurologic findings (Irfan et al., 2021). Among the neurologic symptoms, COVID-19 has been increasingly associated with risk of ischemic strokes (Wijeratne et al., 2020).

Multiple pathogenetic mechanisms underlying this risk have been explicated. These include COVID-19 inducing a hypercoagulable state, and causing vasculopathy and cardiomyopathy. The virus attaches to the angiotensin converting enzyme-2 (ACE-2) receptors allowing host cell entry. Such receptors are ubiquitous and are found in the vascular endothelium and the enterocytes of the small intestines. Upon entry to the host cell, the virus stimulates a cascade of cytolytic immune responses, activating both the cellular and humoral defenses. The interplay of the virus' direct effects and the host's reaction to the infection leads to multiple systemic effects increasing the risk for ischemic strokes (Figure 2) namely – (1) inflammation, (2) coagulopathy, (3) endothelial damage, (4) platelet activation, and (5) vasculopathy (Stein et al., 2021).

### 4.2. Pediatric ischemic strokes in the background of COVID-19

Pediatric ischemic strokes in COVID-19 patients have been reported to occur as AIS or CSVT. In a cross-sectional study of pediatric patients with ischemic strokes, AIS had the greatest number of cases who are positive with SARS-CoV-2 (Beslow et al., 2021). Interestingly, our systematic review is likewise composed of a significantly large fraction of patients with childhood AIS (64/74), four of which had concomitant CSVT. On the other hand, there were nine cases of childhood CSVT and only one case of neonatal AIS.

It has been described that the first week after birth may carry the highest risk for ischemic stroke with incidence similar to that of adults (Lynch and Nelson, 2001; Nelson and Lynch, 2004; Mineyko and Kirton, 2011). The data gathered in our study does not seem to follow this trend. Since COVID-19's incidence in children was noted to be similar across all age groups (Dawood et al., 2022), the clustering of documented ischemic strokes in older children highlights the difference in the pathogenesis of stroke in COVID-19 compared to the commonly identified risk factors during the first week of life (Martinez-Biarge et al., 2016; Sorg et al., 2020). It is also possible that neonates with COVID-19 may have subtle or no clinical manifestations even in the presence of ischemic strokes (Lee et al., 2017), thus resulting in low detection and underreporting.

### 4.3. Inflammation and coagulopathy in COVID-19 and ischemic strokes

The link between the systemic inflammatory response syndrome in COVID-19 and its subsequent increased ischemic stroke risk has been established with evidence of increase in procoagulants (Masi et al., 2020) and frequent clinical documentation of disseminated intravascular coagulation (DIC)-like massive clot formations in COVID-19 patients (Iba et al., 2020). Surge of procoagulants may be secondary to the release of the von Willebrand factor (vWF) from an injured endothelium or from hypoxia-mediated hypercoagulability (Masi et al., 2020) producing an imbalance of the physiologic anticoagulant-procoagulant homeostasis leading to a hypercoagulable state.

Furthermore, there was minimal protraction of prothrombin time (PT) and activated partial thromboplastin time (aPTT) with mild thrombocytopenia in COVID-19 infection suggestive of a mechanism unique from that of the classic sepsis-induced coagulopathy (Langer et al., 2020; Tang et al., 2020). In another study, elevated plasminogen activator inhibitor-1 (PAI-1) and tissue plasminogen activator (tPA) (Zuo et al., 2021) were noted indicating derangements in the fibrinolytic pathways and reflective of the hemostatic system's reaction to a COVID-19-induced thrombophilia.

In our data, 61.8% of the pediatric ischemic stroke with COVID-19 cases have concomitant elevation of inflammatory markers (i.e., ESR, CRP, IL-6, fibrinogen, ferritin, procalcitonin and D-dimer).

Furthermore, D-dimer, both an inflammatory and a thrombophilic marker is specifically elevated in 38% of the cases in our study. Increasing level of this marker has been associated with a worsening inflammatory state and disease severity (Esenwa et al., 2021). These findings in our cohort support the COVID-19-associated coagulopathy (CAC) or thromboinflammation described in previous studies (Connors and Levy, 2020; Levy et al., 2021a).

### 4.4. Endothelial damage, platelet activation and inflammatory vasculopathy

Endothelial cell injury from direct SARS-CoV-2 invasion or indirectly by the acute systemic inflammation has been the proposed mechanism for COVID-19 inducing endotheliopathy and vasculopathy (Hamming et al., 2004). This has been supported by studies showing elevated markers of endothelial injury like PAI-1, vWF, syndecan-1 and thrombomodulin (Dupont et al., 2021). On the other hand, the resulting dysfunctional endothelium contributes to the severity of COVID-19 (Ruhl et al., 2021).

An intact vascular endothelium is inactive and possesses natural anticoagulant properties. Upon damage, it becomes a medium for platelet activation and stimulation of the coagulation cascade. It becomes highly procoagulant providing a nidus for thrombus formation (Levy et al., 2021b). Pediatric cases of cerebral arterial and venous thrombosis (Asif and O'Mahony, 2020; Essajee et al., 2020; Anvekar et al., 2021; Appavu et al., 2021; Beslow et al., 2021; Dakay et al., 2021; de Marcellus et al., 2021; Hadjiu et al., 2021; Jillella et al., 2021; Español et al., 2022) reported in this paper clinically showcased this mechanism.

Inflammation of the arterial vessel wall, particularly of central nervous system (CNS) vasculitis or vasculopathy is likewise reported (Becker, 2020; Vaschetto et al., 2020; Quintas-Neves, 2021; Salihefendic et al., 2021). It is proposed that the endothelial dysfunction could be secondary to the changes in the structural and functional configuration of the endothelial cells which can subsequently lead to arterial stiffness and morphologic changes on the capillaries (Jud et al., 2021). In another study, another vascular wall effect of COVID-19 is the increased risk of atherosclerosis secondary to the inflammatory cascade and the alteration of the ACE-2 activity leading to disrupted renin-angiotensin system (Lou et al., 2021). These vasculopathic effects, particularly in the CNS, were elucidated in almost one-third of the pediatric case reports identified in this study (Gulko et al., 2020; Mirzaee et al., 2020; Appavu et al., 2021; Beslow et al., 2021; de Marcellus et al., 2021; Ellis et al., 2021; Foster et al., 2021; Khosravi et al., 2021; Tiwari et al., 2021; Wilkinson et al., 2021; Poisson et al., 2022).

### 4.5. Cardiac injury and risk of ischemic stroke

Multiple mechanisms have been proposed to cause cardiac damage in the background of SARS-CoV-2 infection. These include cardiac myocyte damage from the high load of cytokines during the systemic inflammation, the direct cell injury brought by SARS-CoV-2 (viral myocarditis), other mechanisms like arrhythmia from electrolyte derangements during the critical phase of the illness, and medications used in the treatment such as steroids, antiviral and immunological therapies (Driggin et al., 2020; Zheng et al., 2020). Decompensation of chronic heart failure and acute coronary syndrome from the inflammatory process and elevated catecholamines were the identified complications in adults (Xiong et al., 2020).

In this pediatric study, prominent cardiac complications were seen mostly in patients who concomitantly have MIS-C presenting as cardiogenic shock. Echocardiographic studies in other patients in this review showed cardiac failure and presence of intracardiac thrombus (cardioembolism) (Kaushik et al., 2021; Khoshnood et al., 2021; Sa et al., 2021; Wilkinson et al., 2021; Chang et al., 2022). This demonstrates that the cardiac complication of COVID-19 is another way of causing an arterial ischemic event in this group of patients.

### 4.6. Comorbidity predisposing to stroke in COVID-19

Multiple risk factors predisposing patients to develop stroke have been established. Among adults with COVID-19, the risk of ischemic stroke has been shown to be higher in the presence of concomitant cardiovascular risk factors such as diabetes mellitus, hypertension, dyslipidemia, heart failure and arrhythmias (Qureshi et al., 2021).

Dehydration, infection, fever, hypoxic-ischemic injury, head injuries and surgeries, anemia and autoimmune conditions are known risk factors that predispose patients to CSVT (Dlamini et al., 2010). On the other hand, risk factors for AIS in the pediatric age group includes cardiac disease, infections, prothrombotic conditions, malignancy, head and neck trauma, autoimmune conditions and genetic disorders (Numis and Fox, 2014).

In this systematic review, identified comorbidities include anemia (Appavu et al., 2021; Beslow et al., 2021; Español et al., 2022) hemoglobinopathies (Beslow et al., 2021; Sa et al., 2021), hematologic malignancies (Sánchez-Morales et al., 2021; Whitworth et al., 2021; Karimi et al., 2022), structural cardiovascular anomalies (Khoshnood et al., 2021; Sánchez-Morales et al., 2021), prior infection with varicella (Beslow et al., 2021), systemic and CNS bacterial and tuberculous infections (Essajee et al., 2020; de Marcellus et al., 2021; Fraser et al., 2022).

**Figure 3:**
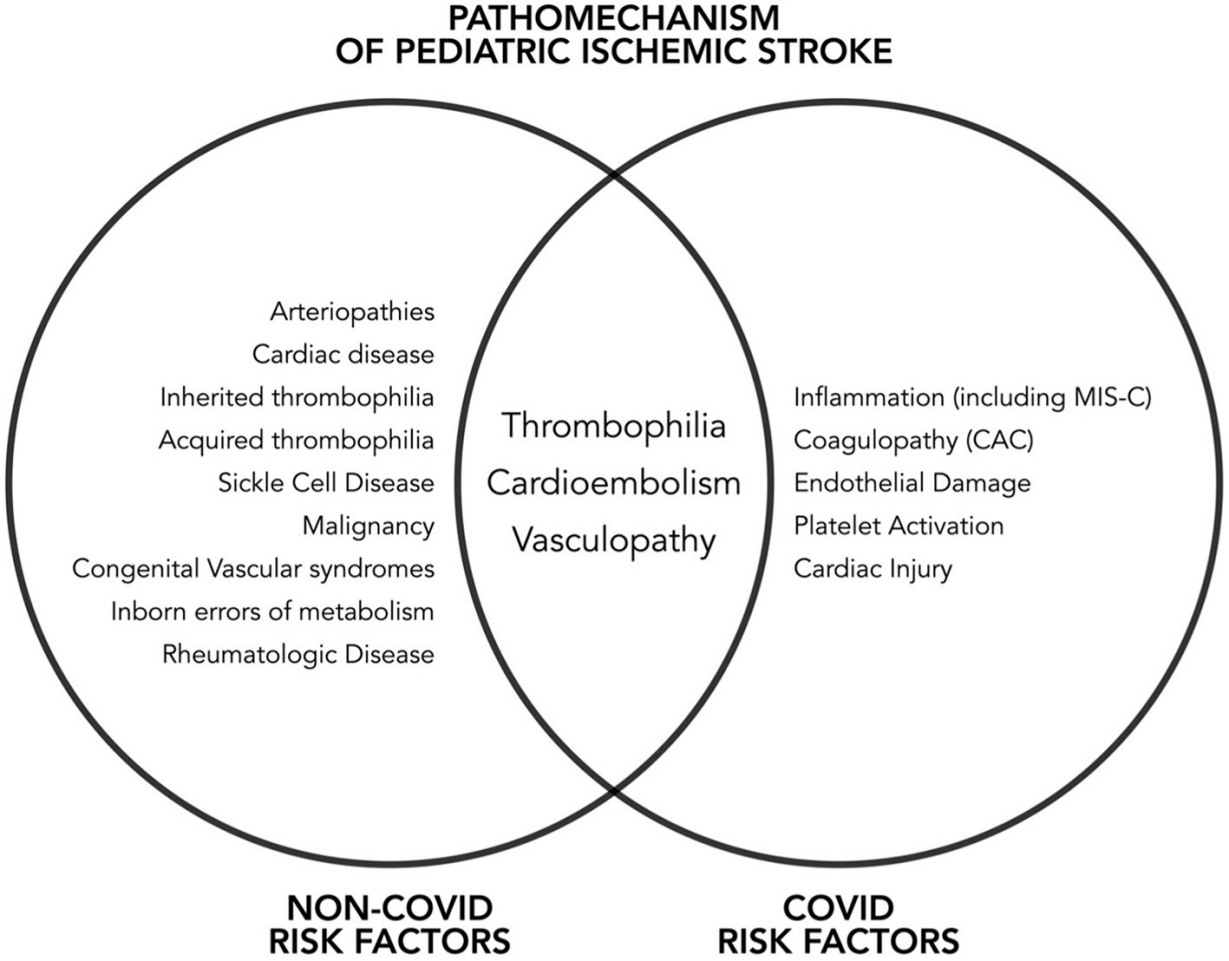
Non-COVID and COVID risk factors for pediatric ischemic stroke.

With the novelty of SARS-CoV-2 and the limited data in childhood strokes, there is no available study yet expounding the possible potentiation of stroke risk by COVID-19 infection in children with comorbid conditions. Given the described COVID-19 pathogenetic mechanisms and the clustering of reported cases with other stroke risk factors, we hypothesize that the presence of such comorbidities may lead to a higher overall predisposition to ischemic strokes (Figure 3).

### 4.7. Treatment options

In this study, we observed that centers utilized COVID-19-recommended strategies in approaching care for their patients. These included supportive management and viral-targeted therapy (remdesivir) (Chiotos et al., 2021). Although data are inconclusive and interim recommendations evolve (WHO, 2022a), we recognized a trend in the use of steroids among severe or critically-ill patients, including those with MIS-C.

Stroke-directed therapies included the use of antiplatelet, anticoagulants, thrombolysis and mechanical thrombectomy. There is still no standardized approach in the management of ischemic stroke in the background of COVID-19. We observed that centers still followed treatment guidelines (Ferriero et al., 2019) intended for non-COVID-19-related pediatric ischemic strokes.

### 4.8. Outcomes

Mortality was observed in 10/58 or 17.24% of cases in articles that reported outcomes and notably half of which were stroke-related. All these stroke-associated mortalities were due to large vessel occlusions (LVOs), mostly involving MCA or MCA-ACA distributions suggesting that involvement of a bigger arterial territory portends a higher risk of mortality. LVOs therefore may reflect a more severe COVID-19 disease.

## 5. Limitations

We acknowledge that the study has multiple limitations. Given the rarity of the disease, the majority of the publications are limited to case reports and series, with one observational cohort. These types of publications are considered to have lower quality and are at risk for reporting bias. Furthermore, there may be reporting bias in publications with preference for those cases with remarkable neuroimaging findings and clinical course. Heterogeneity is anticipated when incorporated studies are mostly case reports or series. Each paper displays relevant variables differently from the other. Hence, these limitations inherent to the publication types available for review restricted the feasibility of deriving generalization using the data. Lastly, language restrictions prevented identification of other possible related case reports.

Identifying the role of comorbid conditions and their effect on the development of ischemic strokes in children with COVID-19 is likewise a limitation of the study as comparing this group to those who did not develop stroke is not within the scope of the study design.

Finally, this review showcases the need for an international registry of pediatric COVID-19 cases with strokes to address the knowledge gap and help in elucidating further the association between COVID-19 and pediatric ischemic strokes with the long term goal of developing evidence based guidelines in the diagnosis and management of these patients.

## 6. Conclusion

Pediatric ischemic stroke is an uncommon but important complication of COVID-19. The interplay between SARS-CoV-2 and the host elicits a complex cascade of reactions that lead to a deranged hemostatic system and vasculopathic changes with a final common pathway resulting in ischemic strokes. Brain and cerebral vessel imaging studies, echocardiograms and inflammatory markers together with routine coagulation studies and thrombophilic work-ups will help determine the etiology of the stroke. Recommended therapies for COVID-19 in children are utilized in this cohort. Adaptation of current treatment guidelines for pediatric ischemic strokes was observed to be useful in pediatric patients with ischemic strokes in the background of COVID-19 but require more extensive and controlled studies in the future.

## Data availability statement

The original contributions presented in the study are included in the article/Supplementary material, further inquiries can be directed to the corresponding author.

## Author contributions

EL and MT: conceptualization, data curation, formal analysis, interpretation of data, writing-original draft, and writing-review and editing. RK: conceptualization, data curation, formal analysis, interpretation of data, and writing-review and editing. AA: conceptualization, data curation, formal analysis, writing-review and editing, and graphical illustration. All authors contributed to the article and approved the submitted version.
