# Supplementary material for: Mechanisms of pediatric ischemic strokes in COVID-19: a systematic review
Source: Front Stroke. 2023 Jul 3;2:1197714. doi: 10.3389/fstro.2023.1197714 (PMC12802804; doi:10.3389/fstro.2023.1197714)
Supplement: Supplementary file 1 [file Table_1.DOCX]

**Supplemental Table 1. List of strategies utilized in database search**

| **Database** | **Search strategy** |
| --- | --- |
| **MEDLINE (through Pubmed)** | ((((((((((((pediatric stroke[MeSH Terms]) OR (childhood stroke[MeSH Terms])) OR (stroke in children[MeSH Terms])) OR (stroke in adolescent[MeSH Terms])) OR (pediatric arterial ischemic stroke[MeSH Terms])) OR (childhood arterial ischemic stroke[MeSH Terms])) OR (arterial ischemic stroke in children[MeSH Terms])) OR (arterial ischemic stroke in adolescent[MeSH Terms])) OR (pediatric cerebral sinus venous thrombosis[MeSH Terms])) OR (childhood cerebral sinus venous thrombosis[MeSH Terms])) OR (adolescent cerebral sinus venous thrombosis[MeSH Terms])) AND (COVID-19[MeSH Terms] OR SARS-CoV-2 virus[MeSH Terms])) |
| **EMBASE** | ('pediatric stroke'/exp OR 'pediatric stroke' OR 'childhood stroke'/exp OR 'childhood stroke' OR 'stroke in children' OR 'stroke in adolescent' OR 'pediatric arterial ischemic stroke' OR 'childhood arterial ischemic stroke'/exp OR 'childhood arterial ischemic stroke' OR 'arterial ischemic stroke in children' OR 'arterial ischemic stroke in adolescent' OR 'pediatric cerebral sinus venous thrombosis' OR 'childhood cerebral sinus venous thrombosis' OR 'adolescent cerebral sinus venous thrombosis') AND ('covid'/exp OR covid) |
| **Web of Science** | “COVID-19” AND “pediatric stroke” OR “pediatric arterial ischemic stroke” OR “childhood cerebral sinus venous thrombosis” OR “pediatric CSVT” |
| **Google Scholar** | “COVID-19” AND “pediatric stroke” OR “childhood stroke” OR “stroke in children” OR “stroke in adolescent” OR “pediatric arterial ischemic stroke” OR “childhood arterial ischemic stroke” OR “arterial ischemic stroke in children” OR “arterial ischemic stroke in adolescent” OR “pediatric cerebral sinus venous thrombosis” OR “childhood cerebral sinus venous thrombosis” OR “adolescent cerebral sinus venous thrombosis” OR “pediatric cerebral sinovenous thrombosis” OR “childhood cerebral sinovenous thrombosis” OR “adolescent cerebral sinovenous thrombosis” OR “pediatric CSVT” OR “childhood CSVT” OR “adolescent CSVT” |
