# Supplementary material for: Mechanisms of pediatric ischemic strokes in COVID-19: a systematic review
Source: Front Stroke. 2023 Jul 3;2:1197714. doi: 10.3389/fstro.2023.1197714 (PMC12802804; doi:10.3389/fstro.2023.1197714)
Supplement: Supplementary file 2 [file Table_2.docx]

**Supplemental Table 2: Demographic data, comorbidities and clinical presentation of COVID-19 patients diagnosed with ischemic stroke**

| **Author** | **Patient**  **Profile** | **Clinical and Neurologic Presentation** | **COVID-19**  **Status** | **MIS-C** | **Other Underlying/ Comorbid Diseases** | **Other Organs and Systems Involved** | **Other Relevant Stroke Work-ups** | **Imaging modality / Type of ischemic stroke / Arterial or Venous Territory** | **Intervention** | **Outcome** |
| --- | --- | --- | --- | --- | --- | --- | --- | --- | --- | --- |
| Beslow et al.(1) | 4 days old / F | No systemic symptoms  No neurologic deficits | PCR (+) | NA | NA | NA | NA | MRI – thalamocapsular infarct | NA | NA |
|  | 28 months/ F | Asymptomatic  Neurologic deficit unspecified | PCR (+)  Ab (+) | NA | Varicella infection 6 months prior | NA | NA | MRI / MRA – L basal ganglia infarct / L MCA stenosis; focal cerebral arteriopathy | NA | NA |
|  | 32 months / F | Three days prior – fever  Neurologic deficit unspecified | PCR (+)  Ab (+) | NA | NA | NA | NA | CT / MRI – midbrain infarct | NA | NA |
|  | 10 years old/ M | Fever, cough, severe respiratory distress  Neurologic deficit unspecified | PCR (+) | NA | Iron deficiency anemia | Pulmonary | NA | CT – bilateral PCA infarcts | NA | NA |
|  | 10 years old / M | Fever, Lower limb thrombosis Neurologic deficit unspecified | PCR (+)  Ab (+) | (+) | Sickle cell disease | NA | NA | CT / MRI –R MCA and cerebellar infarcts  MRA – R ICA narrowing | NA | NA |
|  | 14 years old / M | Fever, cough, headache, diarrhea, severe respiratory distress  Neurologic deficit unspecified | PCR (+) | (+) | Iron deficiency anemia | Pulmonary,  GI | NA | CT – R MCA and R ACA infarcts | ECMO | NA |
|  | 9 years old / M | With mastoiditis  Cough  Neurologic deficit unspecified | PCR (+) | NA | NA | Pulmonary | NA | CT / MRI / MRV – R transverse sinus and R sigmoid sinus thrombosis | NA | NA |
| Ivanova et al. (2) | 16 years old / NA | Severe course with bilateral polysegmental pneumonia, polyserositis, hypoxic encephalopathy | (+) Unspecified Testing | NA | NA | Pulmonary | NA | Superior sagittal, transverse, L sigmoid sinus thrombosis | Thrombolysis | NA |
| Appavu et al.(3) | 8 years old / F | 3 weeks prior with fever and cough  transfused with blood  R hemiplegia and language impairment  NIHSS Score 15 | PCR (-)  Ab (+) | NA | NA | Pulmonary | Anemia Elevated inflammatory markers,  Echocardiography, 12-L ECG, Troponin – Normal  Thrombectomy specimen: platelet and fibrin-rich thrombus with RBCs, WBCs | MRI – bilateral MCA territory infarcts  MRA – proximal L M1 occlusion  VWI – concentric mural enhancement of the L ICA (inflammatory intracranial arteriopathy) | Mechanical thrombectomy  heparin, aspirin, methylprednisolone | On discharge, mRS 4 |
|  | 16 years old / M | 30 days prior – with fever, cough and positive  SARS-CoV-2 PCR test with interim improvement of symptoms;  7-day history of progressive lethargy, limping and decreased responsiveness;  Right hemiparesis and global aphasia  NIHSS Score 19 | PCR (+)  Ab (+) | NA | NA | Pulmonary | Elevated troponin, prolonged PR interval, elevated creatinine and inflammatory markers, elevated D-dimer, fibrinogen and Factor VIII activity, (+) Lupus anticoagulant antibodies | MRI / MRA – complete L MCA infarct, arteritis of L M1 – MCA  VWI 2 weeks post-presentation: no mural enhancement | Heparin | At 2 months follow-up: mRS score 3 |
| de Marcellus et al.(4) | 16 years old / M | Fever, signs of meningeal irritation, stupor, hyperreflexia, eventually with R hemiplegia and aphasia | PCR (+) | NA | Lemierre syndrome, bacterial meningitis | NA | Elevated inflammatory markers;  CSF evidence of bacterial meningitis  (WBC 1566/uL, 76% neutrophils);  Blood culture – *Fusobacterium necrophorum* and *Streptococcus constellatus* | MRI/MRA on admission – vasculitis – stenosis of the L ICA, MCA and ACA with vessel wall enhancement);  MRI after R hemiplegia and aphasia:  L MCA ischemic stroke; L ophthalmic vein thrombosis; worsening vasculitis  CT scan D14: worsening vasculitis involving the basilar artery; Large bilateral infarcts in ACA, MCA territories | Cefotaxime, metronidazole, levofloxacin, dexamethasone, aspirin, enoxaparin, tocilizumab, remdesivir | Death |
| Español et al.(5) | 8 years old / F | One-day history of fever, myalgia and chest pain (acute chest syndrome);  Hypertonic lower extremities on Day 13 hospitalization | PCR (+) | (+) | Hemoglobin SS disease, on hydroxyurea | Pulmonary | Anemia (Hgb 6.7 g/dL), leukocytosis, neutrophilia, lymphopenia;  Elevated inflammatory markers;  Chest radiograph – consolidation and atelectasis, R middle-lower lung zones | Hospital Day 13 CT/MRI/MRA – cortical vein thrombosis with infarction, R posterior parietal region  Hospital Day 21 – resolution of the cortical vein thrombosis with vasculitis of the ACA | IV Fluids, blood transfusion, tocilizumab, remdesivir, methylprednisolone, LMWH, enoxaparin | Discharged after 38 hospital days without neurologic sequelae |
| Anvekar et al.(6) | 12 years old / M | Fever, body aches, cough;  Altered mental status, signs of increased ICP | PCR (+) | NA | NA | Pulmonary | Elevated D-dimer,  Coagulation assays-normal | MRI – R temporoparietal hemorrhagic lesion  MRV – CSVT, sigmoid, lateral, and jugular sinuses | Enoxaparin | Normal neurologic examination on monthly follow-ups |
| Hadjiu et al. (7) | 7 months old / M | Fever, respiratory distress syndrome, bilateral pneumonia;  Bilateral tonic-clonic seizures, L hemiparesis | Ab (+) | NA | NA | Pulmonary | Elevated inflammatory markers | Thrombosis on the transverse and sigmoid sinus, L; with areas of arterial ischemic strokes (acute – subacute supratentorial R > L) and diffuse areas of necrosis and encephalomalacia on the frontoparietal and periventricular regions | Supportive | NA |
|  | 9 months old / M | Fever;  L hemiparesis, involuntary movements on the R extremities | PCR (+)  Ab (+) | NA | NA | NA | Elevated inflammatory markers | Supratentorial ischemic stroke extended acute-subacute bilateral | Supportive | NA |
| Essajee et al.(8) | 2 years old / F | L hemiparesis | PCR (+) | NA | Disseminated (miliary and meningitis) tuberculosis | NA | Elevated D-dimer, fibrinogen and ferritin  Gastric washings – AFB Positive | CT –infarct on the anterior limb of the R internal capsule, thalamus and lentiform nucleus; CSVT | Anti-TB medications  Prednisone  Aspirin  Ventriculoperitoneal shunt insertion | GCS 15, residual L hemiparesis |
| Asif et al.(9) | 18 years old / M | One week prior – fever, cough, myalgia with resolution;  Headache, mild photophobia | PCR (+) | NA | NA | Pulmonary | Normal coagulation profile, elevated CRP | CTV– hyperdensity of the internal cerebral veins / CSVT - sigmoid, transverse sinuses, straight and superior sagittal sinuses | LMWH, enoxaparin | At 2 weeks follow up – marked resolution of the headache |
| Jillella et al.(10) | 12 years old / M | Fever, malaise;  Syncope, dysarthria, aphasia, R hemiparesis | Ab (+) | NA | NA | NA | Echocardiogram – Normal  CRP – normal  Normal extensive evaluation for inherited or acquired hypercoagulable studies | CT – initially normal; on repeat ASPECTS Score 9 (L MCA)  CTA – occlusion of the L supra-clinoid ICA  Angiography – thrombus in the L supra-clinoid ICA | Thrombectomy, aspirin | Discharged improved (mRS score 1) |
| Dakay et al.(11) | 17 years old / M | Two-week history of L-sided headaches, hypertension, vomiting, blurring of vision, and papilledema | PRC (+) | NA | Obesity | NA | Elevated D-Dimer  Elevated lipoprotein A and Factor VIII activity | MRI / MRV – extensive venous thrombosis | Enoxaparin | Discharge to home;  Improving clot burden on repeat imaging |
| Ellis et al.(12) | 13 months old / F | One week prior – rhinorrhea  No other systemic symptoms  R hemiparesis, central facial palsy and hemineglect  NIHSS 5 | Ab (+) | NA | NA | NA | NA | MRI – Acute infarct, L basal ganglia  Angiogram: L MCA-M1 segment – post-infectious vasculopathy | NA | Restoration of baseline function |
| Wilkinson et al.(13) | 12 years old / F | Fever, abdominal pain, cardiac, renal and hepatic dysfunction;  Symmetric bilateral superior hemifield defects | PCR (+)  Ab (+) | (+) | NA | Pulmonary,  Cardiac,  Renal,  GI | Elevated inflammatory markers  MIS-C criteria met | MRI /MRA – ischemic strokes on bilateral PCA and hippocampal arteries  Focal cerebral arteriopathy – (inflammatory) on the proximal P2 segments and bilateral P1 | Enoxaparin, IVIG, anakinra, methylprednisolone, prednisone, aspirin | At 4 weeks follow-up  No improvement of visual deficits |
| Foster et al.(14) | 4 years old / M | No other systemic symptoms;  Headache, vomiting, tonic-clonic seizure;  eventually with L hemiparesis | PCR (+)  Ab (+) | NA | Unspecified congenital anomaly causing hydrocephalus | NA | Echocardiogram – normal  Coagulopathy work-up – Normal  CSF infectious studies – Negative  Elevated CSF proinflammatory markers | CT scan (on admission) – subacute hydrocephalus  MRI (post-EVD) – membrane obstructing R foramen of Monro  MRI / MRA –vasculopathy on the distal L carotid artery, bilateral MCA, R ACA, and bilateral PCA | Steroids  External ventricular drain, septum pellucidotomy | No development of new infarcts with resolution of the vascular findings |
| Poisson et al.(15) | 8 years old / F | Two weeks prior – fever, cough, headache, hyperreflexia, ankle hypertonia, Babinski, L;  With continued neurologic deterioration despite immunotherapy given for the vasculitis;  Secondary MAS developed,  L hemiparesis,  Status epilepticus | PCR (+)  Ab (+) | NA | NA | Pulmonary | NA | MRI – R frontal lobe enhancing lesion with surrounding vasogenic edema  Brain biopsy – Lympho-histiocytic vasculitis (SV-cPACNS) | Methylprednisolone, plasmapheresis;  With vasculitis confirmation:  cyclophosphamide,  rituximab, IVIG, infliximab | Death  unrelenting clinical decline |
| Gulko et al.(16) | 13 years old /F | Two months prior – fever, myalgia, anosmia;  Headache, speech difficulty, R hemiparesis | Ab (+) | NA | NA | NA | Echocardiogram – Normal  CSF studies – Normal including viral panel (HSV, VZV);  Normal inflammatory markers | MRI –infarcts in the L MCA territory  MRA – L M1-MCA focal moderate stenosis  VWI – focal cerebral arteriopathy of childhood (inflammatory) | NA | Neurologically improved at time of examination |
| Tiwari et al.(17) | 9 years old / F | Headache, vomiting, fever, conjunctivitis, hypertension;  GCS 11 (E3V2M6),  R central facial palsy, R hemiplegia, R extensor plantar sign | PCR (-)  Ab (+) | (+) | NA | Pulmonary,  GI | Chest X-Ray –ground-glass and reticulonodular opacities,  Elevated CRP, ESR, D-dimer, triglyceride and ferritin levels  CSF – pleocytosis, with slight increased protein | CT –infarcts in the corpus callosum, L basal ganglia and bilateral thalami  CTA – multifocal stenosis of bilateral intracranial ICA, R MCA, bilateral ACA; diffusely narrowed bilateral MCA | IVIG, methylprednisolone, dexamethasone, remdesivir, LMWH | At 3 weeks – GCS 13 (E4V3M6) with residual R hemiparesis |
| Mirzaee et. al. (18) | 12 years old / M | No other systemic symptoms;  Seizures, R hemiparesis and dysarthria | PCR (+) Ab (+) | NA | NA | NA | ESR elevated,  Normal coagulation parameters,  Echocardiogram– normal  CSF studies – normal,  HSV-1 and 2, VZV – negative | MRI / MIP – infarctions on the L MCA territory / focal irregular narrowing and banding of proximal L M2-MCA with mildly decreased distal flow (focal cerebral arteriopathy) | Conservative management | Residual hemiparesis |
| Khosravi et al.(19) | 10 years old / F | One week prior – low grade fever,  Headache, seizures, dysarthria, L central facial palsy and hemiparesis | PCR (+) | NA | NA | GI | Coagulation profile normal;  Elevated LDH | MRI / MRA – acute infarction in the R basal ganglia and posterior insula / R MCA focal narrowing  MRV – no cerebral venous thrombosis | Enoxaparin, Aspirin, aspirin | Improved and discharged home |
| Kaushik et al(20) | 5 years old/ M | Fever, cough, abdominal pain, emesis, shock, respiratory failure;  Anisocoria (fixed dilated right pupil) on ECMO Day 5 | PCR (-)  Ab (+) | (+) | NA | Pulmonary,  GI | Abdominal X-ray – non-obstructed distended bowel loop pattern  Abdominal and pelvic CT –fluid-filled small intestinal loops  Pro-BNP and Troponin elevated  Elevated inflammatory markers (ferritin, D-dimer, fibrinogen levels)  Elevated cytokine storm panel (CRP, IL-6, Il-8, TNF-alpha)  Echocardiogram – depressed LVEF | CT – R ACA-MCA artery infarctions and L frontal SAH | ECMO  Antithrombin III transfusion,  Tocilizumab  Heparin | Death  brain herniation |
| Khoshnood et al.(21) | 3 years old / F | Fever;  Gait instability,  dysarthria, aphasia | PCR (-)  Ab (+) | (+) | Down syndrome, Congenital heart disease status post-repair | NA | Elevated inflammatory markers  Echocardiogram – large intracardiac thrombus | MRI –infarcts on multiple cerebral cortical regions, L cerebellum; | IVIG, methylprednisolone,  surgery – removal of cardiac thrombus | Improved and discharged home;  with residual neurologic deficits |
| Chang et al.(22) | 15 years old / F | One week prior – headache, fever, abdominal pain and generalized weakness;  Acute aphasia and R hemiparesis (Last known neurologically normal – 10 hours prior)  NIHSS Score 15 | PCR (+) | (+) | NA | Cardiac,  Renal | Elevated inflammatory markers, renal and cardiac dysfunction, leukocytosis  Echocardiogram – LVEF 45%  Cardiac MRI – mild pericardial effusion  (-) Deep vein thrombosis on Doppler | CT / CTA: L insular hypodensity / distal L MCA-M2 branch occlusion  MRI: infarcts in the frontal operculum, insula, L caudate, splenium of the corpus callosum | IVIG, methylprednisolone, prednisone, enoxaparin | PSOM 0 at Day 30 follow-up |
|  | 16 years old / F | Three day fever, nausea, vomiting and diarrhea, hypotension;  R lower extremity DVT;  Obtundation, aphasia and R hemiparesis | Ab (+) | (+) | NA | GI | Cardiac MRI – apical thrombus | CT: L insular hypodensity  Head and neck CTA: L ICA terminus occlusion  MRI: infarcts in the L insula, caudate, frontal, temporal lobes, splenium of the corpus callosum | Heparin  IVIG, methylprednisolone, prednisone | PSOM 2 at Day 41 follow-up (mild expressive aphasia and residual R hemiparesis) |
| Sa et al.(23) | 14 years old / M | Fever, respiratory distress, diarrhea, cardiac dysfunction and hypotension;  Headache,  focal neurologic deficits | PCR (-)  Ab (+) | (+) | Alpha-thalassemia trait, Obesity | Cardiac | Elevated inflammatory markers | CT – Acute infarct, R ACA | No immune therapy given | Death |
|  | 10 years old / M | Fever, sickle cell crisis, abdominal pain, vomiting; Encephalopathy, L hemiparesis, signs of increased ICP | PCR (-)  Ab (+) | NA | Sickle Cell Disease | GI | Elevated inflammatory markers | CT / MRI – R frontal infarction with intraparenchymal hemorrhage | IVIG  Tocilizumab | L hemiparesis (mRS score 4) |
| Sanchez-Morales et al.(24) | 12 years old / M | Decreased sensorium, aphasia, L hemiparesis, seizures | Ab (+) | NA | Aortic coarctation | NA | NA | MRI – acute ischemic stroke in left frontal lobe | NA | Dysphasia with residual weakness, acalculia |
|  | 16 years old / F | L hemiparesis, mixed aphasia | Ab (+) | NA | Acute myeloblastic leukemia | NA | NA | MRI – acute ischemic stroke in watershed areas | NA | Death bacterial sepsis |
| Fraser et al. (25) | 6 months old / NA | Four-day history of cough, congestion and respiratory distress, shock and acidosis, splenic and renal infarctions;  Anisocoria, Left-sided hemiparesis | PCR (+) – Delta variant | NA | Tuberculous meningitis | Pulmonary,  Renal | Chest radiograph – multifocal pneumonia  Elevated inflammatory markers with evidence of disseminated intravascular coagulopathy  Echocardiogram – normal;  *Mycobacterium tuberculosis* growth – lungs, liver and subarachnoid space | MRI / MRA – R MCA and R PCA territory ischemic stroke, punctate left thalamic ischemic stroke / Proximal occlusion of the R MCA and R PCA | Dexamethasone, remdesivir  Anticoagulation – deferred due to hemorrhagic risk | Death from multiorgan failure |
| Kalyani et al.(26) | 12 years old / F | Fever, vomiting;  Seizures; cerebellar deficits | (+) Unspecified Testing | NA | NA | NA | Normal coagulation tests, echocardiogram, carotid vertebral doppler;  ANA test – negative | Initial CT scan – Normal  MRI – Lacunar infarct, R cerebellar hemisphere, R PICA territory | Not specified | NA |
|  | 16 years old / M | R central facial palsy; R upper arm weakness | (+) Unspecified Testing | NA | NA | NA | NA | MRI – Acute Infarct, L lower pons | Antiplatelets, statin, neuroprotective measures, IV fluids | NA |
| Shen et al.(27) | 17 months old / M | Exposure to COVID-19-confirmed parents;  No other systemic symptoms;  Acute R hemiparesis | PCR (-)  Ab (+) | NA | NA | NA | Normal inflammatory, autoimmune, hypercoagulable and multisystem inflammatory syndrome markers  Normal genetic testing  Normal CSF studies (including viral panel)  Echocardiogram – Normal | L pontine ischemic stroke  MRA Head and Neck – Normal | Aspirin | Minimal residual weakness at 3^rd^ month follow-up |
| Kangin et. al.(28) | 7 years old / F | No other systemic symptoms  Hemodynamic compromise, deterioration of sensorium;  Seizures, aphasia,  signs of brain herniation | PCR (+) | NA | NA | NA | Normal D-dimer, CRP, Ferritin, PT, INR, homocysteine | CT / MRI – ischemic stroke, L ACA – MCA territories | Medical decompression with hypertonic saline | Demise |
| Khan et al.(29) | 15 years old / F | Pneumonia,  Seizures, horizontal nystagmus, tongue tremor | PCR (-)  Ab (+) | (+) | NA | Pulmonary | Elevated inflammatory markers  Chest X-ray – ARDS | CT – ischemic infarct, R frontal lobe; small intra-cerebral hematoma, L frontal lobe | Dexamethasone followed by oral steroids | At 4 months follow-up – No neurological deficit |
| Schupper et. al.(30) | 5 years old / M | Fever, cough, abdominal pain, cardiogenic shock;  Developed cardiopulmonary failure requiring ECMO;  Fixed dilated pupils on 5^th^ day ECMO | Ab (+) | (+) | NA | Pulmonary,  Cardiac | Elevated IL-6 | CT – R MCA infarction, edema and contralateral SAH | On Heparin prior to stroke  ECMO | Demise, brain death |
|  | 2 months old / M | Respiratory failure requiring ECMO  Nonconvulsive status epilepticus on four antiseizure medications | PCR (+) | (+) | NA | Pulmonary | Elevated IL-6 | Cranial UTZ – multifocal echogenicity suspicious for hemorrhage  CT – infarcts on bilateral MCA and PCA territories  Interval MRI – hemorrhagic transformation in bilateral occipito-parietal lobes, L frontal and temporal lobes | ECMO | Weaning from ventilator as of publication |
| Kihira et al. (31) | 5 years old / M | Three-day fever, cough and abdominal pain, cardiogenic shock;  Anisocoria on D4 ECMO | PCR (-)  Ab (+) | (+) | NA | Pulmonary  Cardiac | Echocardiogram- Ejection fraction 30%, cardiomegaly;  Chest radiograph – bronchovascular prominence;  Elevated D-dimer | CT – R ACA-MCA infarction with SAH in the L hemisphere | Heparin  ECMO | Demise,  brain herniation |
| Shahi et al.(32) | 17 months / F | Fever, diarrhea, nausea, vomiting;  Focal jerking movements of R upper extremity,  R hemiparesis | PCR (+) | NA | NA | GI | Echocardiogram – normal  CSF – normal, negative Viral panel  Normal work-ups for thrombophilia | CT scan / MRI – Acute L MCA territory infarct  MRA – Normal | Enoxaparin  Aspirin  Phenytoin | Discharged home improved and stable |
| Chesmedzhieva et al.(33) | 8 years old / M | Three-day history of rhinorrhea;  No cough or fever  Headache, L central facial palsy and hemiparesis,  hyperreflexia, positive Babinski and Chaddock, L | PCR (-)  Ab (+) | NA | NA | Pulmonary | NA | MRI – Acute R MCA territory infarct  Cerebral angiography – Occlusion of R MCA | LMWH, shifted to Edoxaban  Citicoline  Piracetam  Mannitol  Dexamethasone | At 3 months follow-up: improved with residual hemiparesis |
| Scala et al.(34) | 11 years old / M | Two weeks prior – rhinorrhea and sore throat;  L hemiplegia,  dysarthria, nystagmus | Ab (+) | NA | NA | Pulmonary | Maternal history – absent Factor V (Leiden), Factor II and MTHFR mutations  Elevated fibrinogen, D-dimer, homocysteine | CT / MRI – R MCA territory infarct, in early stage (DWI/FLAIR mismatch) | Thrombolysis then endovascular thrombectomy; decompressive hemicraniectomy; anticoagulant and antiplatelet | Residual L hemiparesis |
| Swartwood et al.(35) | 16 years old / M | No other systemic symptoms  L hemiparesis, L facial palsy,  L-sided visual neglect,  dysarthria | Ab (+) | NA | NA | NA | Echocardiogram – PFO; no thrombus  Mildly elevated D-dimer  Inflammatory markers – unremarkable  CSF studies – normal | CT – R MCA thrombus  CTA (post-thrombolysis) – residual thrombus, R MCA | tPA at 2 hours from onset  Aspirin  Supportive measures (IV Fluids, neuroprotective measures) | Significant resolution with deficits |
| Pabst et al.(36) | School-aged / F | Fever, abdominal pain, throat swelling, vomiting, myalgia, and hyperemic conjunctivae;  3^rd^ day of admission -  L hemiplegia, L facial palsy, homonymous hemianopsia, dysarthria  PedNIHSS 16 | PCR (+) | (+) | NA | GI,  Renal | Transaminitis, renal failure, hyponatremia, elevated inflammatory markers, increased D-dimer and troponin levels  Echocardiogram – no thrombus  Cardiac MRI – no myocarditis | MRI - Acute R lenticulostriate infarct; occlusion of R MCA-M1 segment | Thrombectomy  Aspirin  IVIG  Methylprednisolone  Enoxaparin | PedNIHSS 1 on discharge |
| Abdalla et al.(37) | 2 years old / F | Fever, rhinorrhea and cough;  Generalized seizures, deterioration of sensorium, hypertonia and hyperreflexia, pinpoint reactive pupils | PCR (+) | NA | Malaria | Pulmonary | Normal hematologic and coagulation parameters  Normal electrolytes  Unremarkable liver profile | CT – bilateral basal ganglia infarct | Acyclovir | GCS 11 on discharge |
| Keskin et al.(38) | 9 years old / M | Five-day history of fever, gastrointestinal symptoms, respiratory distress and shock;  Post-stroke venothromboembolism while on prophylactic enoxaparin;  L hemiparesis, L central facial palsy | Ab (+) | (+) | NA | Pulmonary,  GI | Echocardiogram – LVEF 51%  Elevated inflammatory markers | MRI – infarct on the R temporal lobe and sylvian fissure | Enoxaparin | D 28 Follow-up – residual mild L hemiparesis |
| Silvestri et al.(39) | 15 years old / M | No other systemic symptoms  Dysarthria, paresthesia of right arm and cheek, headache, vomiting, deterioration of sensorium, stiff neck | PCR (+) | NA | NA | NA | Elevated CRP, Fibrinogen, D-Dimer  Normal hypercoagulable work-up (proteins C and S, homocysteine, mutations in prothrombin, Factor V Leiden, MTHFR genes) | MRI – CSVT: cortical veins, superior sagittal sinus; subacute ischemic areas in centrum semiovale, right corona radiata | Enoxaparin  Dexamethasone  Aspirin | 1 month follow-up: partial resolution of the CSVT |
| Wadhawan et al.(40) | 2 years old / F | No other systemic symptoms  Sudden-onset R hemiparesis | Ab (+) | (+) | NA | NA | Echocardiogram – LVEF 30-35% (myocarditis)  Elevated inflammatory markers, D-dimer, and NT-proBNP levels | MRI – Acute L MCA infarct | Enoxaparin  Aspirin  Furosemide  Spirinolactone | 6 months follow-up – minimal gliosis on previous region of infarction |
| Szydlowski et al.(41) | 14 years old /M | No other systemic symptoms  Nystagmus, diplopia, abnormal eye movements, anisocoric pupils | PCR (+) | NA | NA | NA | Homozygous MTHFR 677C>T mutation,  Normal Leiden,  Thrombophilic work-up (Homocysteine, Protein C and S, D-dimer) -normal,  Echocardiogram – LVEF 70%, hypokinesis on the apical region  Cardiac MRI – LV and LA thrombus formation on the surface of myxoma | MRI – Acute infarct, L posterior thalamus | Aspirin  Nadroparin  Cardiac surgery for thrombus and myxoma removal | 9 days MRI post-follow up  Resolution of ischemia |
| Riphagen et al.(42) | 14 years old / M | 4 day history of fever, headache, diarrhea and abdominal pain, respiratory distress;  Deterioration of sensorium, shock | PCR (+) | NA | NA | Pulmonary, GI | Echocardiogram – RV dysfunction, elevated RV systolic pressure  Elevated inflammatory markers (Ferritin, CRP, procalcitonin)  Elevated D-dimer, proBNP  Thrombocytopenia | Neuroimaging – R MCA and ACA ischemic infarction | IVIG  Hydrocortisone | Demise |
| Saini et al. (43) | 12 years old/ F | 5 weeks prior – had COVID-19 infection with no respiratory involvement  Weakness of the right upper limb | (+)  Not specified | NA | NA | NA | Normal inflammatory markers | MRI - Ischemic stroke  Normal VWI | Aspirin | On admission:  mRS 3  On 3 months follow-up:  mRS 0 |
|  | 8 years old/ F | 4 weeks prior – had COVID-19 infection  With no respiratory involvement  Right hemiparesis | (+)  Not specified | NA | NA | NA | Elevated inflammatory markers | MRI - Ischemic stroke  MRA/VWI – focal cerebral arteriopathy, A1 segment of R ACA, M1 segment of the L MCA | Steroids  Aspirin | On admission:  mRS 4  On 3 months  follow-up:  mRS 0 |
| Shobhavat et al.(44) | 3 years old/ F | Neurologic deficit, not specified | NA | NA | NA | NA | NA | Subacute white matter infarct with radial artery thrombi | LMWH | NA |
| Whitworth et al.(45) | 12-14 years old/ M | Neurologic deficit, not specified | NA | NA | Cancer, unspecified | NA | NA | Cerebral sinovenous thrombosis | Anticoagulant, unspecified | NA |
|  | 16-18 years old/ M | 1 month prior – diagnosed with COVID-19  Critically ill  Neurologic deficit, not specified | (+)  Not specified | (+) | NA | NA | NA | Acute MCA stroke | No thromboprophylaxis given | NA |
| Spanelova et al.(46) | 1 year old/ F | No other systemic symptoms  Sudden onset incomplete external ophthalmoplegia with ptosis | PCR (-)  Ab (+) | NA | Factor V Leiden heterozygous and increased Factor VIII | NA | NA | VWI – arterial wall enhancement of Left P1-PCA  Cytotoxic edema of left mesencephalon with bleed in the prepontine cistern | Pulse doses of corticoids shifted to prednisone for 3 months  LWMH  Aspirin | Mild ptosis |
|  | 11 years old/ F | Repeated COVID-19 infection presenting with colds, middle ear infection and protanopia – last infection 1 month prior to ictus;  Acute right upper extremity paresthesia, facial palsy and dysarthria that resolved spontaneously after 5 minutes | PCR (-)  Ab (+) | NA | NA | Pulmonary | NA | CT/CTA – negative  MRA/DWI - negative  MRI – lacunar infarct, L basal ganglia | Aspirin | Normal |
|  | 4 years old/ M | Headache, vomiting, fever, signs of meningeal irritation, papilledema and seizures | PCR (-)  Ab (+) | NA | NA | NA | Severe thrombocytopenia, elevated D-dimer | MRI – hemorrhagic venous infarction due to L CVST passing into the L IJV | Decompressive hemicraniectomy  LMWH  Steroids  IVIG | Hemiparesis, palsy and dysarthria |
| Osman et al.(47) | 5 years old/ M | Systemic symptoms of fever, rash gastrointestinal symptoms and conjunctivitis;  Acute dysarthria, facial palsy and hemiparesis with headache irritability and decreased sensorium | Ab (+) | NA | NA | NA | NA | MRI – ischemic infarct, L MCA | Dexamethasone  Ceftriaxone / Acyclovir | With residual deficits |
| AlKandari et al.(48) | 15 years old/ F | Fever;  Facial palsy, right hemiparesis and hemihypesthesia | PCR (+) | NA | NA | NA | Elevated D-dimer | MRI – acute infarct, pons and L cerebellum  CTA – occlusion of the L vertebral and basilar arteries, thrombotic in nature (MRI) | Aspirin  Clopidogrel | Partial motor recovery |
| Avital et al.(49) | 14 months old/ F | 2 months prior – with 2 day history of fever, headache, sore throat, ageusia and loss of smell;    Had a 5-10 min episode of gait disturbance and left hemiparesis that resolved spontaneously which recurred and persisted thereafter | PCR (-)  Ab (+) | NA | NA | NA | Normal inflammatory markers | CT - R MCA ischemic stroke  CTA – severe stenosis in the M1 of the R MCA | Nimodipine  Methylprednisolone  Aspirin | Normal ambulation with minimal weakness of the L upper extremity |
| Grigore et al.(50) | 17 years old/ F | Rhinorrhea, cough, diarrhea, fatigue;  Intermittent right-sided headaches, nausea and vomiting | PCR (+) | NA | NA | Pulmonary,  GI | NA | MRI-MRA- CSVT on the R sigmoid sinus, transverse sinus bilaterally, confluence of the transverse sinuses and the R IJV | LMWH followed by  Acenocoumarol | Resolution of headaches, nausea and vomiting  6^th^ months follow-up: MRA normal venous flow |
| Karimi et al.(51) | 5 years old/ F | Fever,  Seizures | PCR (+) | NA | precursor B-cell ALL,  Febrile Neutropenia,  Vincristine Chemotherapy | NA | Bilateral ground glass opacities on Lung CT | CT –ischemic stroke, R parietal | Phenytoin  Levetiracetam  Remdesivir  Vitamin C, D, A  Subcutaneous enoxaparin for 2 months | At 3 months follow-up: With moderate motor and speech deficits  At 10 months – full recovery of neurologic deficits |
| Vielleux et al.(52) | 3 years old/NA | Encephalopathy, R hemiparesis, preferential gaze to L | PCR (+)  Ab (+) | (+) | NA | NA | NA | L M2 ischemic stroke | Heparin | Mild R hemiparesis |
|  | 10 years old/ F | R hemiparesis, neglect and preferential gaze to L | PCR (-)  Ab (+) | (+) | NA | NA | NA | L M1 ischemic stroke | tPA  Thrombectomy  ASA | No residual deficits |
|  | 11 years old/NA | R hemiparesis, dysarthria and headache | PCR (-)  Ab (+) | NA | NA | NA | NA | Ischemic stroke of the L basal ganglia from bilateral ICA dissection | ASA | Mild R hemiparesis and spasticity |
|  | 12 years old/NA | BL superior hemianopia | PCR (+)  Ab (+) | (+) | NA | NA | NA | BL PCA territory ischemic stroke from FCA | Prednisone  ASA | Persistent neurologic deficits |
|  | 16 years old/NA | R hemiparesis and dysarthria | PCR (+) | NA | NA | NA | NA | Acute ischemic stroke, L medial lenticulostriate artery from primary angiitis | Prednisone | Mild weakness of the R lower extremity |
|  | 16 years old/NA | L hemiparesis and dysarthria | PCR (+) | NA | NA | NA | NA | R MCA embolic stroke | tPA  ASA | Mild weakness of L lower extremity |

*List of Table Abbreviations:* Ab - Antibody; ACA – Anterior Cerebral Artery; ALL – Acute Lymphoblastic Leukemia; ARDS – Acute Respiratory Distress Syndrome; ASA – Aspirin; ASPECTS – Alberta Stroke Programme Early CT Score;; BL – Bilateral; COVID-19 – Coronavirus Disease 2019; CRP – C-Reactive Protein; CSVT – Cerebral Sinovenous Thrombosis; CTA – Computed Tomography Angiography; CT – Computed Tomography; CXR – Chest X-Ray; D – Day; DWI- Diffusion weighted imaging; DVT – Deep Vein Thrombosis; ECG – Electrocardiography; ECMO – Extracorporeal Membrane Oxygenation; ESR – Erythrocyte Sedimentation Rate; EVD- external ventricular drainage; F – Female; FCA – Focal Cerebral Arteriopathy; FLAIR- Fluid attenuated inversion recovery; GCS- Glasgow Coma Scale; GI – Gastrointestinal; GM – CSF – Granulocyte Macrophage – Colony Stimulating Factor; HSV – Herpes Simplex Virus; ICA – Internal Carotid Artery; ICP – intracranial pressure; IJV – Internal Jugular Vein; IL – Interleukin; INR – International Normalized Ratio; IV- intravenous; IVIG – Intravenous Immunoglobulin; L – Left; LDH – Lactate Dehydrogenase; LMWH – Low Molecular Weight Heparin; LVEF – Left Ventricular Ejection Fraction; M – Male; MCA – Middle Cerebral Artery; MAS – Macrophage Activation Syndrome; MIS-C - Multisystem Inflammatory Syndrome in Children; MRA – Magnetic Resonance Angiography; MRI – Magnetic Resonance Imaging; MRV – Magnetic Resonance Venography; MTHFR - Methylenetetrahydrofolate reductase; mRS – modified Rankin Scale; NA – Not Applicable; NIHSS – National Institute of Health Stroke Scale; NPS – Nasopharyngeal Swab; PedNIHSS - Pediatric National Institute of Health Stroke Scale; PCA – Posterior Cerebral Artery; PCR – Polymerase Chain Reaction; PFO – Patent Foramen Ovale; PICA – Posterior Inferior Cerebellar Artery; PSOM- Pediatric stroke outcome measure; PT – Prothrombin Time; R – Right; RBC – Red Blood Cell; RV – Right Ventricle; SAH – Subarachnoid Hemorrhage; SARS-CoV-2 – Severe Acute Respiratory Syndrome Coronavirus 2;SV-cPACNS - Small-Vessel Primary Angiitis of the CNS in Childhood; TNF-alpha – Tumor Necrosis Factor – Alpha; tPA – Tissue Plasminogen Activator; UTZ – Ultrasound; VWI – Vessel Wall Imaging; VZV – Varicella Zoster Virus; WBC – White Blood Cell

References:

1. Beslow, L. A., Linds, A. B., Fox, C. K., Kossorotoff, M., Zuñiga Zambrano, Y. C., Hernández-Chávez, M., et al. (2021). Pediatric ischemic stroke: an infrequent complication of SARS-CoV-2. Ann. Neurol. 89, 657–65. doi: 10.1002/ana.25991
2. Ivanova, A. A., Shamsheva, O. v., and Shchederkina, I. O. (2021). Study Q12 of the role of an infectious factor in the development of stroke in children. Results of a 5-year retrospective analysis. Children Infections. 20, 10–5. doi: 10.22627/2072-8107-2021-20-2-10-15
3. Appavu, B., Deng, D., Dowling, M. M., Garg, S., Mangum, T., Boerwinkle, V., et al. (2021). Arteritis and large vessel occlusive strokes in children after COVID-19 infection. Pediatrics. 147, e2020023440. doi: 10.1542/peds.2020- 023440
4. de Marcellus, C., Dupic, L., Roux, C. J., el Aouane, E. l., Ghomari, I., Parize, P., et al. (2021). Case report: cerebrovascular events associated with bacterial and SARS-CoV-2 infections in an adolescent. Front. Neurol. 12, 479. doi: 10.3389/fneur.2021.606617
5. Español, M. G., Gardner, R. v., Alicea-Marrero, M. M., Marrero-Rivera, G., Bradford, T., LeBlanc, D. M., et al. (2022). Multisystem inflammatory syndrome in a pediatric patient with sickle cell disease and COVID-19: a case report. J. Pediatr. Hematol. Oncol. 44, E134–7. doi: 10.1097/MPH.0000000000002191
6. Anvekar, P., Lohana, P., Kalaiger, A. m., Ali, S. R., and Galinde, R. S. (2021). The unfamiliar case of COVID-19 induced cerebral venous sinus thrombosis in a pediatric patient. Cureus. 13, e17209. doi: 10.7759/cureus.17209
7. Hadjiu, S., Calcii, C., Sprincean, M., and Revenco, N. (2021). Accidental vascular Q19 cerebral in infectia COVID-19 La Copii. Buletin de perinatologie. 2, 69−73.
8. Essajee, F., Solomons, R., Goussard, P., and van Toorn, R. (2020). Child with tuberculous meningitis and COVID-19 coinfection complicated by extensive cerebral sinus venous thrombosis. BMJ Case Rep. 13, 238597. doi: 10.1136/bcr-2020-238597
9. Asif, R., and O’Mahony, M. S. (2020). Rare complication of COVID-19 presenting as isolated headache. BMJ Case Rep. 13, e239275. doi: 10.1136/bcr-2020-239275
10. Jillella, D. v., Philbrook, B., Ortolani, E., Grossberg, J. A., Stani, T., Samuels, O., et al. (2021). Successful endovascular therapy in COVID-19 associated pediatric ischemic stroke. J. Stroke Cerebrovasc. Dis. 30, 106152. doi: 10.1016/j.jstrokecerebrovasdis.2021.106152
11. Dakay, K., Cooper, J., Bloomfield, J., Overby, P., Mayer, S. A., Nuoman, R., et al. (2021). Cerebral venous sinus thrombosis in COVID-19 infection: a case series and review of the literature. J. Stroke Cerebrovasc. Dis. 30, 105434. doi: 10.1016/j.jstrokecerebrovasdis.2020.105434
12. Ellis R, Hartman K, Barndt B, Francio V. Pediatric Post-infectious Ischemic Vasculopathic Stroke: A Potential Neurological Complication Associated with COVID-19 Infection. PM R. 2021; 13(S1) (suppl 1)
13. Wilkinson, S. W., Etheridge, T., Swiston, C. J., Vegunta, S., Wiggins, R. H., Warner, J. E. A., et al. (2021). Bilateral posterior cerebral artery stroke from COVID-related multisystem inflammatory syndrome in a child. J. Neuroophthalmol. doi: 10.1097/WNO.0000000000001468
14. Foster, C. H., Vargas, A. J., Wells, E., Keating, R. F., and Magge, S. N. (2021). Cerebral vasculopathy and strokes in a child with COVID-19 antibodies: illustrative case. J. Neurosurg. 2, CASE21160. doi: 10.3171/CASE21160
15. Poisson, K. E., Zygmunt, A., Leino, D., Fuller, C. E., Jones, B. v., Haslam, D., et al. (2022). Lethal pediatric cerebral vasculitis triggered by severe acute respiratory syndrome coronavirus 2. Pediatr. Neurol. 127, 1–5. doi: 10.1016/j.pediatrneurol.2021.11.003
16. Gulko, E., Overby, P., Ali, S., Mehta, H., Al-Mufti, F., Gomes, W., et al. (2020). Vessel wall enhancement and focal cerebral arteriopathy in a pediatric patient with acute infarct and COVID-19 infection. AJNR Am. J. Neuroradiol. 41, 2348. doi: 10.3174/ajnr.A6778
17. Tiwari, L., Shekhar, S., Bansal, A., and Kumar, S. (2021). COVID-19 associated arterial ischaemic stroke and multisystem inflammatory syndrome in children: a case report. Lancet Child Adolesc. Health. 5, 88–90. doi: 10.1016/S2352-4642(20)30314-X
18. Mirzaee, S. M. M., Gonçalves, F. G., Mohammadifard, M., Tavakoli, M., and Vossough, A. (2020). Focal cerebral arteriopathy in a pediatric patient with COVID-19. Radiology. 297, E274–5. doi: 10.1148/radiol.2020202197
19. Khosravi, B., Moradveisi, B., Abedini, M., Behzadi, S., and Karimi, A. (2021). Stroke in a child with SARS-CoV-2 infection: a case report. eNeurological Sci. 23, 100345. doi: 10.1016/j.ensci.2021.100345
20. Kaushik, S., Ahluwalia, N., Gangadharan, S., Esperenza, M., Murthy, R., OforiAmanfo, G., et al. (2021). ECMO support in SARS-CoV2 multisystem inflammatory syndrome in children in a child. Perfusion. 36, 524–8. doi: 10.1177/0267659120954386
21. Khoshnood, M., Mahabir, R., Shillingford, N. M., and Santoro, J. D. (2021). Post-infectious inflammatory syndrome associated with SARS-CoV-2 in a paediatric patient with down syndrome case report. BMJ Case Rep. 14, 240490. doi: 10.1136/bcr-2020-240490
22. Chang, J., Bulwa, Z., Breit, H., Cherian, L. J., Conners, J. J., Song, S. Y., et al. (2022). Acute large vessel ischemic stroke in patients with COVID- 19–related multisystem inflammatory syndrome. Pediatr. Neurol. 126, 104. doi: 10.1016/j.pediatrneurol.2021.09.013
23. Sa, M., Mirza, L., Carter, M., Carlton Jones, L., Gowda, V., Handforth, J., et al. (2021). Systemic Inflammation Is Associated With Neurologic Involvement in Pediatric Inflammatory Multisystem Syndrome Associated With SARS-CoV-2. Neurol. Neuroimmunol. Neuroinflamm. 8, e999. doi: 10.1212/NXI.0000000000000999
24. Sánchez-Morales, A. E., Urrutia-Osorio, M., Camacho-Mendoza, E., RosalesPedraza, G., Dávila-Maldonado, L., González-Duarte, A., et al. (2021). Neurological manifestations temporally associated with SARS-CoV-2 infection in pediatric patients in Mexico. Childs Nerv. Syst. 37, 2305–2312. doi: 10.1007/s00381-021- 05104-z
25. Fraser, S., Ellsworth, M., Perez, N., Hamilton, H., Fletcher, S., Brown, D., et al. (2022). Cerebral infarctions in an infant with COVID-19 delta variant infection and disseminated tuberculosis. Pediatr. Neurol. 126, 112–3. doi: 10.1016/j.pediatrneurol.2021.10.014
26. Kalyani, J. P., Saravanan, S., Rachel, P., Narayanan, S., and Ravi, P. S. (2022). Case series: 2 post covid young strokes-12 and 16 years. *IOSR Journal of Dental and Medical Sciences.* 21 (02), 2022, 09-13. doi: 10.9790/0853-2102080913
27. Shen, M. Y., Dugue, R., Maldonado-Soto, A. R., Thakur, K. T., Zyskind, I., Vargas, W. S., et al. (2021). Acute ischemic stroke in a pediatric patient with Q12 known exposure to COVID-19 and positive serology. Pediatr. Neurol. 116, 39–40. doi: 10.1016/j.pediatrneurol.2020.12.003
28. Kangin, M., Talay, M. N., Kavak, S., Alparslan, C., Sayinbatur, B., Akar, A., et al. (2022). Brain death in a child as a result of COVID-19-associated acute stroke: the first Q12 case. J. Paediatr. Child Health. 58, 170–2. doi: 10.1111/jpc.15421
29. Khan, A., Chakravarty, A., Jain, A., Harish, R., Naqishbandi, R., Ishani, T., et al. (2021). Clinical spectrum of neurological manifestations in pediatric COVID-19 Q12 illness: a case series. J. Trop. Pediatr. 67, fmab059. doi: 10.1093/tropej/fmab059
30. Schupper, A. J., Yaeger, K. A., and Morgenstern, P. F. (2020). Q12 Neurological manifestations of pediatric multi-system inflammatory syndrome potentially associated with COVID-19. Childs Nerv. Syst. 36, 1579–80. doi: 10.1007/s00381-020-04755-8
31. Kihira, S., Morgenstern, P. F., Raynes, H., Naidich, T. P., and Belani, P. (2020). Fatal cerebral infarct in a child with COVID-19. Pediatr. Radiol. 50, 1479. Q12 doi: 10.1007/s00247-020-04779-x
32. Shahi, M. V., Yousefzadegan, S., Mahmoudabadi, R. Z., Ahmadi, F., and Riahi, A. Q12 (2021). Is a brain stroke caused by COVID-19 seen under two years of age? a case report. Turk J. Neurol. 27, 46–8. doi: 10.4274/tnd.2021.42492
33. Chesmedzhieva, B., and Stanev, S. (2022). Ischemical Stroke In A Child And Its Q18 Association With Covid-19 – Case Report. Knowledge-International Journal
34. Scala, M. R., Spennato, P., Cicala, D., Piccolo, V., Varone, A., Cinalli, G., et al. (2022). Q12 Malignant cerebral infarction associated with COVID-19 in a child. Childs Nerv. Syst. 38, 441–445. doi: 10.1007/s00381-021-05273-x
35. Swartwood, S., Nelson, G. R., and Espinoza, A. C. (2021). Stroke as presenting Q12 feature of COVID-19 in a pediatric patient. J. Pediatr. Neurol. doi: 10.1055/s-0041- Q18 1731396
36. Pabst, L. M., Zyck, S. A., and Youssef, P. (2022). Successful thrombectomy in a pediatric patient with large vessel occlusion and COVID-19 related multisystem Q18 inflammatory syndrome. Interv. Neurol. doi: 10.1177/15910199221080873
37. Abdalla, Y. A., Shaban, M. A. A., Ahmed, K. A. H. M., Haroun, M. S., Eljack Q12 MohMah, F., Eltom, K. S., et al. (2022). Bilateral basal ganglia infarction and bilateral thalamic lesions in sudanese pediatric patient with COVID-19 and malaria coinfection, a case report. Clin. Case Rep. 10, e05322. doi: 10.1002/ccr3.5322
38. Keskin, H., Keskin, F., Yildirim, E., Saritas, S., Polat, G., Colak, A., et al. (2022). Case of venous thromboembolia under enoxaparin prophylaxis after recovering from Q12 acute ischemic stroke in consequence of COVID-19-related MIS-C. J. Pediatr. Infect. Dis. doi: 10.1097/INF.0000000000003488
39. Silvestri, P., Clemente, A., Spalice, A., Febbo,A., Matera, L.,Accardo, F., et al. (2022). Case report: cerebral venous sinus thrombosis in a young child with SARS-CoV-2 Q12 infection: the Italian experience. Front. Neurol. 13, 579. doi: 10.3389/fneur.2022.861345
40. Wadhawan P, Chavan B, Patil S. Stroke with Myocarditis: A Different Post-COVID Syndrome. *Indian J Pediatr*. 2022;89(4):416. doi:10.1007/s12098-021-04028-5
41. Szydłowski, L., Gruszczynska, ´ K., Kusa, J., Stanek, P., Machnikowska-Sokołowska, M., Morka, A., et al. (2022). The unusual history of stroke due to coagulopathy caused Q12 by SARS-CoV-2 infection in a 14-year-old boy with two heart tumors. Kardiologia Polska. 80, 101–2. doi: 10.33963/KP.a2021.0084
42. Riphagen, S., Gomez, X., Gonzalez-Martinez, C., Wilkinson, N., and Theocharis, Q12 P. (2020). Hyperinflammatory shock in children during COVID-19 pandemic. Lancet. 395, 1607–1608. doi: 10.1016/S0140-6736(20)31094-1
43. Saini, L., Krishna, D., Tiwari, S., Goyal, J. P., Kumar, P., Khera, D., et al. (2022). PostQ12 COVID-19 immune-mediated neurological complicationsin children: an ambispective study. Pediatr. Neurol. 136, 20–7. doi: 10.1016/j.pediatrneurol.2022.06.010
44. Shobhavat, L., Solomon, R., Rao, S., Bhagat, I., Prabhu, S., Prabhu, S., et al. (2020). Multisystem inflammatory syndrome in children: clinical features and management— Q12 intensive care experience from a pediatric public hospital in western India. Indian J. Crit. Care Med. 24, 1089–94. doi: 10.5005/jp-journals-10071-23658
45. Whitworth, H., Sartain, S. E., Kumar, R., Armstrong, K., Ballester, L., Betensky, M., et al. (2021). Rate of thrombosis in children and adolescents hospitalized with Q23 COVID-19 or MIS-C. Blood. 138, 190–198. doi: 10.1182/blood.2020010218
46. Španelová, ˇ K., Skríšovská, T., MuŽlayová, P., Horák, O., Šenkyrík, J., Seehofnerová, A., et al. (2022). Cerebrovascular complications of COVID- Q12 19 disease in children: a single-center case series. Pediatr. Neurol. 134, 18–24. doi: 10.1016/j.pediatrneurol.2022.06.007
47. Osman RS, Dawood SS, Thawer SP, et al. SARS-CoV-2 precipitating a stroke in a child? A case report from Tanzania. *Pan Afr Med J*. 2022;42:33. Published 2022 May 12. doi:10.11604/pamj.2022.42.33.33018
48. AlKandari, S., Prasad, L., Al Shabrawy, A. M., Gelbaya, S. A. (2023). Post Q12 COVID-19 ischemic stroke in a 15-Year-old patient. Neurosciences. 28, 62–5. doi: 10.17712/nsj.2023.1.20220064
49. Avital, D., Peretz, S., Perlow, E., Konen, O., Inbar, E., Bulkowstein, Y., et al. (2022). Clinical improvement of a toddler with COVID-19 focal cerebral arteriopathy Q12 possibly due to intra-arterial nimodipine. Eur. J. Paediatr. Neurol. 40, 40–3. doi: 10.1016/j.ejpn.2022.07.007
50. Grigore, I., Miron, I., Gavrilovici, C., Lupu, V. V., Antal, D. C., Schreiner, T. G., et al. Q12 (2023). SARS-CoV-2 Possible Etiology of Cerebral Venous Thrombosis in a Teenager: Case Report and Review of Literature. Viruses. 15, 405. doi: 10.3390/v15020405
51. Karimi, H., Sarmadian, R., Gilani, A., Salajegheh, P., Nejad Biglari, H., and Gholizadeh, M. (2022). Cerebrovascular accident in a child with precursor B-cell acute lymphoblastic leukemia and coronavirus disease 2019: a case report. J. Med. Case Rep. Q23 16, 452. doi: 10.1186/s13256-022-03672-5
52. Vielleux, M. J., Swartwood, S., Nguyen, D., James, K. E., Barbeau, B., Bonkowsky, J. L., et al. (2022). SARS-CoV-2 Infection and Increased Risk for Pediatric Stroke. Pediatr. Q12 Neurol. doi: 10.1016/j.pediatrneurol.2022.10.003
